# Mathematical Modeling of Thin-Layer Drying Kinetics of Tomato Peels: Influence of Drying Temperature on the Energy Requirements and Extracts Quality

**DOI:** 10.3390/foods12203883

**Published:** 2023-10-23

**Authors:** Mihaela Popescu, Petrica Iancu, Valentin Plesu, Costin Sorin Bildea, Fulvia Ancuta Manolache

**Affiliations:** 1 Department of Chemical and Biochemical Engineering, National University of Science and Technology POLITEHNICA Bucharest, 1 Gh. Polizu Street, Building A, Room A056, 011061 Bucharest, Romania; mihaela.popescu@chimie.upb.ro (M.P.); v_plesu@chim.upb.ro (V.P.); sorin.bildea@upb.ro (C.S.B.); 2 National Research and Development Institute for Food Bioresources, 014192 Bucharest, Romania; fulvia.manolache@bioresurse.ro

**Keywords:** β-carotene, drying energy requirements, drying kinetics, lycopene, mathematical modeling, tomato peel hot-air drying

## Abstract

Tomato drying implies high energy consumption due to the high moisture content, and limiting drying temperatures is necessary to avoid carotenoid degradation. To explain the mechanism of moisture transport through the material and to scale up the drying process, drying experiments are needed and supported by mathematical modeling. For the Rila tomato peel drying process, ten thin-layer mathematical models were formulated based on experimental data for six temperatures (50–75 °C) and validated by statistical analysis. Considering the slab geometry of the peels sample and Fick’s second law of diffusion model, the calculated effective moisture diffusivity coefficient values D_eff_ varied between 1.01 × 10^−9^–1.53 × 10^−9^ m^2^/s with *R*^2^ higher than 0.9432. From the semi-theoretical models, Two-term presents the best prediction of moisture ratio with the highest *R*^2^ and lowest χ^2^ and RMSE values. Using the experimental data on extract quality (carotenoid content), two degradation models were formulated. Increasing the drying temperature from 50 °C to 110 °C, a degradation of 94% for lycopene and 83% for β-carotene were predicted. From the energy analysis, a specific energy consumption of 56.60 ± 0.51 kWh is necessary for hot-air drying of 1 kg of Rila tomato peel at 50 °C to avoid carotenoid degradation.

## 1. Introduction

Tomatoes are a nutritional food source in many diets all over the world and are known to provide a number of health benefits. This is mostly because they contain carotenoids such as lycopene and β-carotene, which have antioxidant properties and protect against heart disease and other health conditions [1,2,3,4,5,6]. Tomatoes supply more than 85% of the lycopene required for human nutrition since they are widely consumed in both fresh and tomato-related products [7]. The absorption of lycopene in the body takes place through the diet, and only a small part of it, 10–30%, is assimilated. Therefore, in order to benefit from this compound antioxidant activity, it is recommended to use it as a food additive or supplement [8]. Currently, lycopene-containing functional foods, supplements, and cosmetics are in high demand. Thus, researchers are focused on finding new natural sources, wastes, or methods for lycopene extraction to increase the recovery of this pigment at the industrial level [9]. Natural β-carotene recovered from tomatoes is also important for human nutrition, and it is in high demand in the food supplements market [10]. Lycopene and β-carotene are mostly located in tomato skin, especially in the pericarp, where they are found in chromoplasts [11,12,13]. Thus, the peels are the main part responsible for the carotenoid content of tomatoes.

Tomatoes possess a high moisture content of more than 90%, which makes them susceptible to deterioration after harvesting. Drying is the most popular technique to preserve for longer periods and prevent tomatoes from spoiling due to their high moisture content [5,6,14,15]. Drying allows for substantial weight and volume reductions, lowering packing and shipping costs. Also, it allows product storage and prevents the occurrence of the degradation biochemical reactions caused by the high moisture of the samples [1,16]. Additionally, the vegetable samples are dried and ground before being subjected to extraction in order to optimize extraction yields by increasing the sample-solvent contact surface and facilitating the mass transfer of the components from the sample [3].

There are many different ways to dry tomatoes, and the final product’s quality will vary depending on the variety of tomatoes, the drying rate, the air humidity, the size, the shape, and the thickness of the tomato part subjected to drying, the air velocity, the air temperature, and the efficiency of the drying process [16,17]. The main aspect followed in the drying processes of tomatoes is to avoid the oxidative degradation and isomerization reactions of the carotenoids of interest, lycopene and β-carotene. These reactions are mainly caused by the exposure to light, heat, and oxygen because carotenoids present unsaturated double bonds [9,18]. Moreover, it was observed that dried tomato samples have higher lycopene and β-carotene contents than fresh samples due to the improvement of the extraction process caused by the reduction of the sample moisture [2].

Convective hot-air drying is the most used preservation technique at the commercial level. In this method, the drying temperature, velocity, and time are the only factors that may affect the quality of the dried sample [6,8,19]. This method is successfully applied on tomatoes due to the superior preservation of dried sample quality reflected by the color and carotenoid content [18] and the low cost of operation compared to other methods such as freeze-drying, vacuum oven drying, or oven drying [10,17,20,21]. Different tomato samples were subjected to hot-air drying as tomato slices [18,19,22,23,24], peels [8,21] or pomace [20]. Several final moisture contents of the dried tomato samples were presented in the literature as 4% [25], 5% [26], 6% [12], 6.2% [27], 10–15% [22], 17% [8], and 20% [18]. However, the drying temperature and time need to be properly chosen in order to keep the quality of the final dried product according to the type of tomato sample.

To understand the mechanism of moisture transport through the solid and to scale up the drying process, researchers have shown that drying experimental studies results must be accompanied by mathematical modeling. This is necessary in order to determine the drying kinetics, the energy requirements, and the behavior of the moisture content of the sample in different conditions regarding the drying time, temperature, or the drier characteristics [15,28,29,30,31,32]. Thin-layer drying physical models are mostly used to describe the moisture removal in time from tomato samples as slices [14,19,23,33], pomace [31], or peels [8]. During the drying process, the moisture transported by diffusion is modeled either by Fick’s second law of diffusion model [8,19,32,33] or by other models derived from the analogy of mass transfer with the heat transfer mechanism. One model is Newton’s model, which considers that the changes in time of a property (moisture ratio) are proportional to the driving force of mass transfer (difference of concentration). Appropriate models that describe the drying behavior of tomato samples, mostly of tomato slices, were reported as Page [5,14,31], Modified Page [23], Henderson and Pabis [14,29,31], Midili [31,32], and Two-term [29]. For tomato peel drying, analyzed models were Newton, Page, Henderson and Pabis, Peleg, and Weibull [8].

The aim of this study was to formulate and validate by experiments the drying kinetic models for tomato peels of the Rila variety to predict the best drying temperature and the impact on food quality. Six hot-air drying temperatures in the range of 50–75 °C were investigated, keeping the same final moisture of peels (6–7%). By fitting the experimental moisture ratios, different thin-layer mathematical models derived from Fick’s second law of diffusion model were investigated to predict kinetic parameters and validated by statistical analysis. The effect of the drying temperature on the energy consumption was checked. Also, two degradation models in terms of lycopene and β-carotene contents were presented to evaluate the effect of tomato peel drying temperature on the extract quality.

## 2. Materials and Methods

### 2.1. Reagents

Acetone and hexane used in this study are of analytical grade, purchased from Sigma-Aldrich, Darmstadt, Germany.

### 2.2. Preparation of Tomato Peels

Ripe tomatoes from the Rila variety were acquired from a local Bucharest market. Tomatoes were farmed in Colibasi, Giurgiu County, Romania. Fresh tomatoes were washed using distilled water to remove dirt traces and peeled by hand. Fresh tomato peels were gently dabbed with an absorbent material (paper towel) to remove the excess water. Eighteen fresh tomato peel samples of 30 g were placed in plastic bags and stored at –20 °C until drying [21].

### 2.3. Drying Method

Hot-air drying experiments of tomato peels were carried out in a food dehydrator (Hendi Profi Line, model 229026) at temperatures of 50 °C, 55 °C, 60 °C, 65 °C, 70 °C and 75 °C. For each drying experiment, a weight of 30 g of peels was placed evenly on a drier tray, forming a parallelepiped with around 10 mm thickness, and then they were weighed using an analytical balance (Shimadzu Corporation Berlin, Germany, model AW 220) to determine the initial sample mass subjected to drying. The samples were placed in the dehydrator, where the drying temperature was kept constant. After each 60 min, the sample was weighed, and the peel aspect was monitored. These steps were repeated until the final moisture content of the sample exceeded around 6–7%. Dried tomato peel samples were placed in plastic bags and stored in the freezer at –20 °C until extraction. Each drying experiment was performed in triplicate.

### 2.4. Energy Consumption

The specific energy consumptions E (kWh/kg peels) in the drying experiments performed using the hot-air drying method and fresh tomato peels at six different drying temperatures between 50–75 °C were determined using Equation (1) [33]:

(1) $$E=\frac{A_{sample}\times w_{air}\times \rho_{air}\times Cp_{air}\times \Delta T}{m_{sample}}\times t$$

where A_sample_ is the area of the sample plate (m^2^), w_air_ is the air velocity (m/s), ρ_air_ is the air density (kg/m^3^), Cp_air_ is the specific heat of the air (kJ/kg/°C), ∆T is the temperature difference between the hot air and the ambient (°C), m_sample_ is the mass of the dried peels subjected to drying (kg), while t is the drying time (h).

### 2.5. Moisture Equations

The moisture of tomato peel samples was calculated based on experimental measurements to evaluate the effect of the drying temperatures. The wet-basis moisture M_WB_ (%wt.) of the samples was determined using Equation (2), while the dry-basis moisture M (kg_water_/kg_dried peels_) was calculated with Equation (3):

(2) $$M_{WB}=\frac{m_{w}}{m}\times 100$$

(3) $$M=\frac{m_{w}}{m_{s}}$$

where m_w_, m, and m_s_ represent the water mass from the sample, the mass of the sample, and the mass of the solid in the sample (g), respectively. Further, the moisture ratio MR (dimensionless) of the peels during drying was calculated with Equation (4):

(4) $$MR=\frac{M-M_{e}}{M_{0}-M_{e}}=\frac{M}{M_{0}}$$

where M, M_0,_ and M_e_ represent the dry basis moisture content at any time t, the initial and the equilibrium moisture contents (kg_water_/kg_dried peels_), respectively. The equilibrium moisture content value is small compared to M and M_0_; thus, the error involved in the simplification is neglected [19].

### 2.6. Modelling of Tomato Peels Drying

For fruits and vegetables drying, two types of models, theoretical and semi-theoretical models, were used to predict the moisture behavior over time in a thin layer of wet solid material exposed to a hot air stream. The dominant mechanism of moisture removal is diffusion, and the rate of diffusion is influenced by the moisture content of the material and its porosity. Semi-theoretical models consider the external resistance to mass transfer of moisture between the solid and the hot air, while theoretical models take into account also the internal resistance to mass transfer [28,31]. Moreover, semi-theoretical models are known to provide more accurate results and predictions of the behavior of the drying process than theoretical models that include too many assumptions, which results in a large number of errors [28]. Thin-layer models are mathematical equations that relate moisture changes during drying processes to particular parameters, such as the drying constant or the dimensionless lag factor, which take into account the combined effects of a variety of transport phenomena occurring during drying [8,14,19,30].

#### 2.6.1. Theoretical Diffusion Model

**Figure 1:**
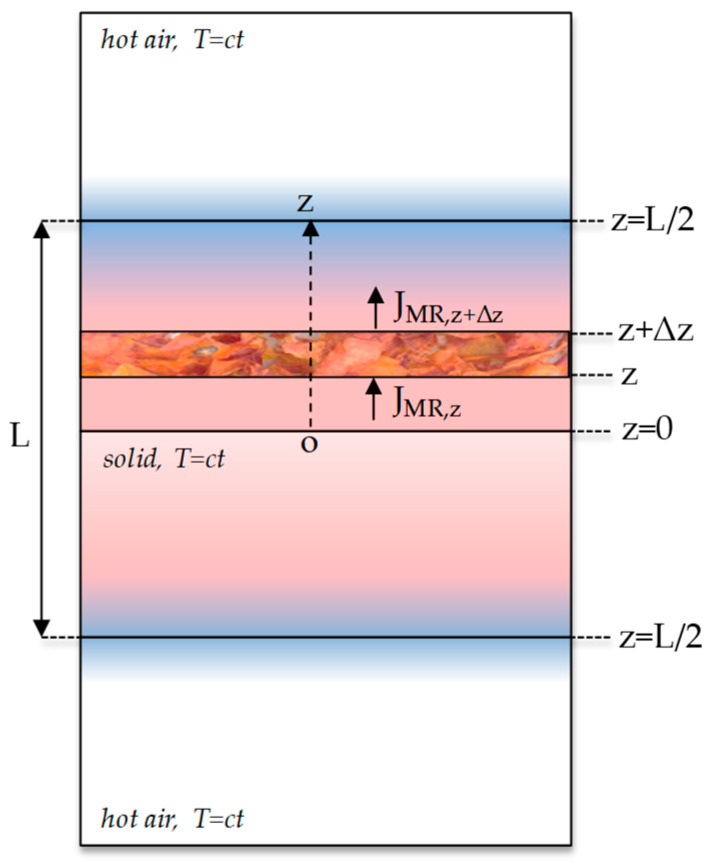
Diffusion physical model at time t > t_0_ (L—the slab thickness (m); J—the moisture mass flux (kg/m^2^/s); z—the diffusion direction; T—the drying temperature (°C)).

**Diffusion physical model.** Based on the theory of diffusion in a plane sheet presented by Crank [34], the physical model of moisture diffusion for tomato peel samples was considered in Figure 1.

The tomato peel bed in the form of a regular parallelepiped slab with geometric characteristics as the cross-sectional area A and the thickness L is placed in a hot air environment in isothermal conditions (T = ct). The moisture diffuses from the center of the slab to the surface in both top and bottom directions (z-axis). At the initial moment (t = t_0_), the moisture (M_0_) is maximum, then when the diffusion starts (t > t_0_), the water concentrates on the surface, and the core of the slab becomes dry. Inside the tomato peel bed, an element of volume with the thickness Δz is considered. The moisture flux entering in this volume is ${\left.J\right|}_{z}$, while the moisture flux leaving the volume is ${\left.J\right|}_{z+\Delta z}$. The diffusion is one-dimensional because there is a concentration gradient only along the *z*-axis [34].

**Diffusion mathematical model**. The model assumptions consider that (a) the moisture is uniformly distributed into the solid sample; (b) there is negligible external resistance for mass transfer; (c) isothermal conditions for convective drying with hot air; (d) the shrinkage effect of the solid during drying is neglected; and (e) the moisture concentration at both slab surfaces are equal. The moisture concentration C_w_, (kg/m^3^) is defined as the moisture amount m_w_ (kg) under the control volume V (m^3^), Equation (5), while the changing of moisture concentration in the control volume ΔC_w_, during time Δt (s) is defined by Equation (6):

(5) $$C_{w}=\frac{m_{w}}{V}$$

(6) $$\Delta C_{w}=\frac{\Delta m_{w}}{A\times \Delta z}$$

where A is the cross-sectional area (m^2^), and ∆z is the thickness (m). The amount of moisture ∆m_w_ that is transported in the control volume in time Δt is defined as the difference between the moisture flux at z + Δz, ${\left.J\right|}_{z+\Delta z}$ (kg/m^2^/s) and the moisture flux at z, ${\left.J\right|}_{z}$(kg/m^2^/s), crossing the area A:

(7) $$\Delta m_{w}={\left.m_{w}\right|}_{z+\Delta z}-{\left.m_{w}\right|}_{z}={\left.J\right|}_{z+\Delta z}\times A\times \Delta t-{\left.J\right|}_{z}\times A\times \Delta t$$

Substituting (7) in (6), the moisture concentration is described by Equation (8):

(8) $$\Delta C_{w}=\frac{{\left.J\right|}_{z+\Delta z}\times A\times \Delta t-{\left.J\right|}_{z}\times A\times \Delta t}{A\times \Delta z}$$

Further, dividing (8) by Δt, the Equation (9) is obtained:

(9) $$\frac{\Delta C_{w}}{\Delta t}=\frac{{\left.J\right|}_{z+\Delta z}-{\left.J\right|}_{z}}{\Delta z}$$

When Δz → 0 and Δt → 0, Equation (9) becomes:

(10) $$\frac{\partial C_{w}}{\partial t}=\frac{\partial J}{\partial z}$$

The flux of water which diffuses through the control volume, in the z direction can be described by Fick’s first law of diffusion, Equation (11):

(11) $$J=-D_{eff}\times \frac{\partial C_{w}}{\partial z}$$

where D_eff_ is the effective moisture diffusivity coefficient (m^2^/s). Equation (10) can be written as:

(12) $$\frac{\partial \left(m_{w}/V\right)}{\partial t}=\frac{\partial }{\partial z}\left[-D_{eff}\times \frac{\partial \left(m_{w}/V\right)}{\partial z}\right]$$

If the diffusivity coefficient D_eff_ and the volume V are constant, and Equation (12) is divided by m_s_ and M_0_, Equation (13) of Fick’s second law of diffusion for a one-dimensional system is obtained:

(13) $$\frac{\partial MR}{\partial t}=-D_{eff}\times \frac{\partial^{2}MR}{\partial z^{2}}$$

The initial condition in the slab, Equation (14), and boundary conditions inside the slab, Equation (15), and at the slab surface, Equation (16), are:

(14) $$t=0;0\leq z\leq \frac{L}{2};MR(z,t)=1$$

(15) $$t>0;z=0;\frac{\partial MR}{\partial z}=0$$

(16) $$t>0;z=\frac{L}{2};MR(z,t)=\frac{M_{e}}{M_{0}}$$

The initial condition describes the uniform distribution of the moisture in the slab, M_0_; the boundary condition at the surface is M_e_ at any time t, while in the core of the slab, there is no diffusion (mass flux is zero) at any time t > t_0_. The analytical solution for the diffusion, Equation (17), is given for the above initial and boundary conditions and for the considered assumptions in the falling rate period and proposed slab geometry of the tomato peels as a sum of infinite Fourier series [34]:

(17) $$MR=\frac{8}{\pi^{2}}\times \left[e^{-\frac{\pi^{2}\times D_{eff}\times t}{4\times {\left(L/2\right)}^{2}}}+\frac{1}{9}\times e^{-\frac{9\times \pi^{2}\times D_{eff}\times t}{4\times {\left(L/2\right)}^{2}}}+\frac{1}{25}\times e^{-\frac{25\times \pi^{2}\times D_{eff}\times t}{4\times {\left(L/2\right)}^{2}}}+\ldots \right]$$

where $\frac{D_{eff}\times t}{{\left(L/2\right)}^{2}}$ is the Fourier number (dimensionless). For long drying times, the Fourier number is higher than 0.2, and only the first term is significant; thus, Equation (17) can be simplified in (18) form without affecting the accuracy of the model’s prediction [32]:

(18) $$MR=\frac{8}{\pi^{2}}\times e^{-\frac{\pi^{2}\times D_{eff}\times t}{4\times {\left(L/2\right)}^{2}}}$$

In logarithmic form, this is an equation of a straight line, with $\left(-\frac{\pi^{2}\times D_{eff}}{4\times {\left(L/2\right)}^{2}}\right)$ slope and $\ln\left(\frac{8}{\pi^{2}}\right)$ intercept, where the slope of the regression line can be used to calculate the value of D_eff_:

(19) $$\ln(MR)=\ln\left(\frac{8}{\pi^{2}}\right)-\frac{\pi^{2}\times D_{eff}}{4\times {\left(L/2\right)}^{2}}\times t$$

In many studies for fruits or vegetables drying, it was presented that the diffusivity coefficient increases with temperature increase, following an Arrhenius relationship:

(20) $$D_{eff}=D_{0}\times e^{-\frac{E_{a}}{R\times T}}$$

where D_0_ is the pre-exponential factor in the Arrhenius equation (m^2^/s), E_a_ is the activation energy (kJ/mol), R is the universal gas constant (8.3145 kJ/mol/K), and T is the drying temperature (K). The activation energy E_a_ is determined from the slope $\left(-\frac{E_{a}}{R}\right)$ of logarithmic form of Equation (20) with $\ln\left(D_{0}\right)$ as the intercept.

(21) $$\ln\left(D_{eff}\right)=\ln\left(D_{0}\right)-\frac{E_{a}}{R\times T}$$

#### 2.6.2. Semi-Theoretical Diffusion Models

For fitting the experimental drying data, semi-theoretical models were also chosen to describe the drying behavior of tomato peels. These models are divided into two categories: semi-theoretical models derived from Newton’s law of cooling (STM–N) and semi-theoretical models derived from Fick’s second law of diffusion (STM–F), as presented in Table 1.

STM–N models consider that the mass transfer by diffusion occurs in the direction of decreasing moisture concentration, similar to heat transfer, which occurs in the direction of decreasing temperature. Based on the analogy between mass transfer by diffusion and heat transfer, the drying rate is defined as proportional to the difference between the moisture at time t and the moisture at equilibrium, Equation (22):

(22) $$\frac{dM}{dt}=-k\times (M-M_{e})$$

with initial condition M = M_0_ at time t = 0 and the solution *MR = e^–k·t^*, where *k* is the drying constant (s^−1^). This equation of Newton Model [35] and other different derived forms obtained by adding one term, such as Page Model [36] or Modified Page Model [37], were used in this work to evaluate the hot-air drying behavior of tomato peels.

STM–F models calculate the moisture ratio using one exponential term, such as the Henderson and Pabis Model [38], many exponential terms, such as the Modified Henderson and Pabis Model [39], Two-term Model [40], and Two-term exponential Model [41], or exponential and linear terms, such as the Midili Model [42] and Logarithmic Model [43]. Two types of constants are considered in these models: the drying constants k, k_1_, k_2_, g, and h (s^−1^) and the empirical constants a, b, c, and n (dimensionless). The higher the number of constants, the more complex the model, although the model efficiency is not necessarily influenced by the number of constants [28].

**Table 1:** Thin-layer mathematical models for the hot-air drying of tomato peels.

Model Type	Model Equation *	Reference
**Theoretical Diffusion Model**		
Fick’s second law of diffusion	MR = 8/π^2^exp(–(π^2^D_eff_t)/4/(L/2)^2^)	[34]
**STM–N Models**		
Newton	MR = exp(–kt)	[35]
Page	MR = exp(–kt^n^)	[36]
Modified Page	MR = exp(–(kt)^n^)	[37]
**STM–F Models**		
Henderson and Pabis	MR = aexp(–kt)	[38]
Modified Henderson and Pabis	MR = aexp(–kt) + bexp(–gt)+ cexp(–ht)	[39]
Midili	MR = aexp(–kt^n^) + bt	[42]
Logarithmic	MR = aexp(–kt) + b	[43]
Two-term	MR = aexp(–k_1_t) + bexp(–k_2_t)	[40]
Two-term exponential	MR = aexp(–kt) + (1 – a)exp(–kat)	[41]

* MR—the moisture ratio (dimensionless); D_eff_—the effective moisture diffusivity coefficient (m^2^/s); L—the slab thickness (m); k, k_1_, k_2_, g, h—the drying constants (s^−1^); a, b, c, n—the model constants (dimensionless); t—the drying time (s).

### 2.7. Carotenoid Extraction Method

The procedure for Soxhlet extraction from tomato peels is structured in three parts: the preparation of the tomato peel sample cartridge, the extraction, and the evaporation of the solvent [21]. Before extraction, the tomato peels were ground for 30 s using a grinder (Tarrington House, model KM150S). A mass of 5 g of dried and ground tomato peels were used for each experiment. Acetone/hexane (*v*/*v*, 1:1) was used as the extraction solvent, and the extraction time was 6 h. The extracts were monitored during the entire extraction time until the solvent from the extractors was discolored, and the extract from the bottom flask became more colorful. For the removal of the solvent, a rotary evaporator (Hahnvapor, model HS-2000NS) was used. The obtained extracts were further transferred in Eppendorf tubes and stored in the freezer at −20 °C until analysis. The results were expressed as extraction yield (g extract/100 g dried or fresh sample). Each Soxhlet extraction experiment was performed in triplicate.

### 2.8. Carotenoids Quantification Method

For lycopene and β-carotene identification and quantification in tomato peel extracts, the UV–*VIS* spectrometry method was applied using a Helios UV–Visible spectrophotometer (Helios beta, Thermo Spectronic). Spectra of tomato peel extracts were recorded in 325–575 nm range using 1 mg of extract diluted in 10 mL acetone/hexane (*v*/*v*, 1:1). Lycopene and β-carotene concentrations in the tomato peel extracts were calculated with the IPM–II–WG_6_ method [44] using absorbances of the samples and absorption coefficients determined from lycopene and β-carotene standard curves at the isosbestic point (461 nm) and at the maximum absorption peak of lycopene (504 nm) in acetone/hexane (*v*/*v*, 1:1). The results were expressed as lycopene (mg lycopene/100 g dried peel) and β-carotene (mg β-carotene/100 g dried peel) concentrations.

### 2.9. Statistical Analysis

**Experimental data analysis**. Drying and extraction experiments, along with the UV–VIS analyses of the extracts for carotenoid quantification, were carried out in triplicate, and the results were reported as mean value ± standard deviation (SD). ANOVA analysis was performed on all the experimental data sets to identify the variability within and between the samples. The drying data of tomato peels, extraction yields, and carotenoid concentrations of the extracts were validated using Hartley’s Fmax test [45] to check the homogeneity of variances for individual sets.

**Validation of the drying mathematical models**. To select the most appropriate fitting model and its accuracy for tomato peel drying using hot air, three statistical parameters were evaluated: the coefficient of determination (R^2^), the reduced chi-square (χ^2^), and the root mean square error (RMSE) using Equations (23)–(25):

(23) $$R^{2}=1-\frac{\sum {\left(MR_{predicted}-MR_{experimental}\right)}^{2}}{\sum {\left({\bar{MR}}_{predicted}-MR_{experimental}\right)}^{2}}$$

(24) $$\chi^{2}=\frac{\sum {\left(MR_{experimental}-MR_{predicted}\right)}^{2}}{N-n}$$

(25) $$RMSE=\sqrt{\left[\frac{1}{N}\sum {\left(MR_{predicted}-MR_{experimental}\right)}^{2}\right]}$$

where MR_experimental_ and MR_predicted_ are the experimental and predicted moisture ratios determined with proposed drying models, N is the number of observations, and n is the number of constants from the model equations [15,32]. The goodness of fitness of the models is associated with high values of R^2^ and low values of χ^2^ and RMSE [15,23,28,29,31].

## 3. Results and Discussion

### 3.1. Experimental Drying Results

**Figure 2:**
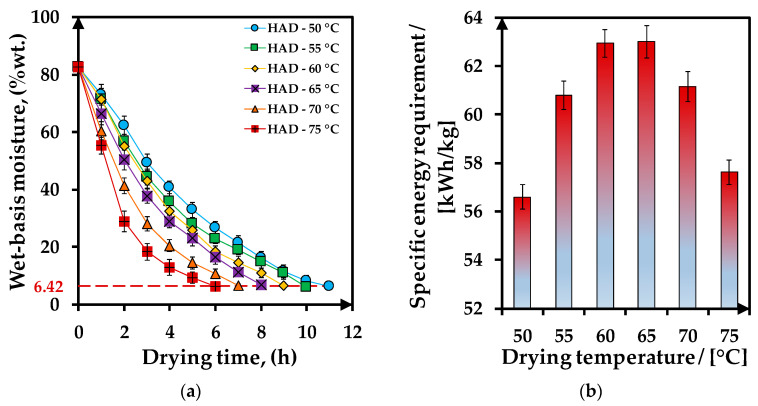
Tomato peels hot-air drying (HAD) characteristics variation with the drying temperature: (**a**) wet-basis moisture; (**b**) specific energy requirements. There were no statistically significant differences between the wet-basis moisture sets and specific energy requirements according to Hartley’s Fmax test (*p* < 0.05) at the α = 0.05 level of significance.

Figure 2a illustrates the variation of the moisture content of the tomato peels during hot-air drying (HAD) at six different temperatures in the 50–75 °C range until each sample reaches a final average wet-basis moisture of around 6.42 ± 0.30%wt., which corresponds to a final moisture ratio of 0.014 ± 0.001. To avoid carotenoid degradation during the drying process of tomato peels, the moisture of the sample should be less than 10% [46] and higher than 4.6% [47]. The initial average wet-basis moisture of used tomato peels was 82.63 ± 1.51%, in the range reported by other authors as 79.13% [8], 80% [25] or 80–85% [48]. As can be seen, the drying time increases from 6 to 11 h with a HAD temperature increase of 5 degrees in the 50–75 °C range for a similar final moisture of the dried tomato peel sample. However, the drying temperature influence is higher than that of the drying time in moisture removal because at higher drying temperatures, the drying time is shorter. At half of the drying time, for temperatures between 50–65 °C, 64.90 ± 0.85% of the total amount of water was removed, while at higher temperatures, the water removal was faster at 70.62 ± 1.75% at 70 °C and 77.83 ± 1.98% at 75 °C. Other studies also reported that at higher temperatures, the dehydration of the sample is increased [5].

Figure 2b presents the variation of the specific energy requirement for tomato peels hot-air drying with the drying temperatures. The specific energy requirement values varied between 56.60 ± 0.51–63.00 ± 0.67 kWh/kg peels, with a minimum value of 50 °C. The drying-specific energy is influenced by the temperature-time combination. The decrease of the specific energy requirement after the temperature of 65 °C may be associated with the decrease in the drying time because, at temperatures of 70 °C and 75 °C, the drying time is 7 and 6 h, respectively.

### 3.2. Determination of Effective Moisture Diffusivity and Activation Energy

The effective moisture diffusivity coefficient D_eff_ values were obtained by fitting experimental data at six different drying temperatures from the slope of linear regression of the falling rate period moisture ratio data and slab thickness of 10 mm (Equation (19)). For tomato peels drying, calculated D_eff_ values varied between 1.01 × 10^−9^–1.53 × 10^−9^ m^2^/s in the drying temperature range of 50–75 °C, increasing with the drying temperature. These values are in the range of 10^−11^–10^−8^ m^2^/s reported in the literature for fruits and vegetables [28], and consistent with the values obtained with different sample thickness: for the drying of tomato peels at 40–70 °C as 1.70 × 10^−9^–12.21 × 10^−9^ m^2^/s [8], and for the drying of tomato slices at 40–60 °C as 0.98 × 10^−10^–6.36 × 10^−9^ m^2^/s [19], at 50–70 °C as 2.53 × 10^−8^–5.00 × 10^−8^ m^2^/s [5], and at 38–64 °C as 3.07 × 10^−9^–6.79 × 10^−9^ m^2^/s [49].

**Figure 3:**
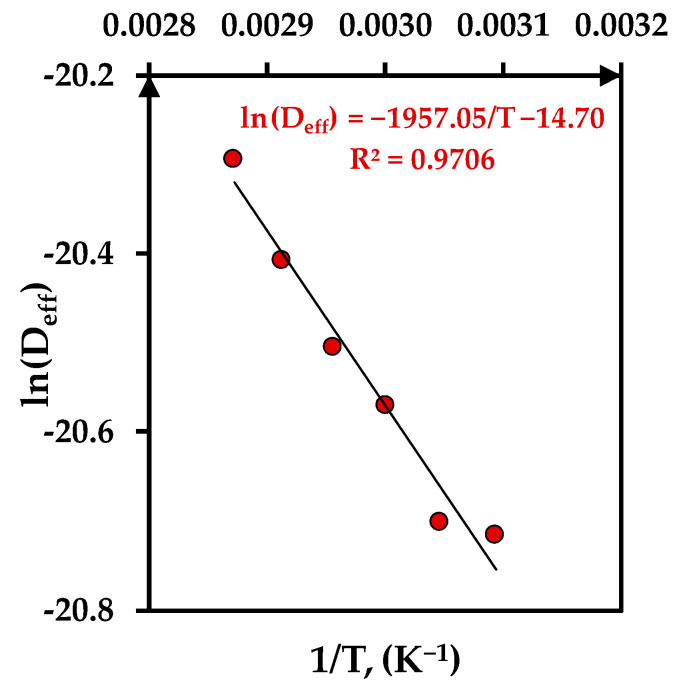
Estimation of the pre-exponential factor D_0_ and activation energy E_a_ for moisture diffusivity coefficient.

The pre-exponential factor and the activation energy for effective moisture diffusivity coefficient at each temperature were determined using Equation (21). Figure 3 presents the linear relationship between ln(D_eff_) and the inverse of the drying temperature (1/T) that presents a high correlation with an R^2^ value of 0.9706 and low values of χ^2^ and RMSE as 0.0010 and 0.0259, respectively.

The estimated value of E_a_ for HAD drying of tomato peels is 16.27 kJ/mol and falls within the general range of 14.42–43.26 kJ/mol reported for drying of fruits and vegetables [28]. Also, the inclusion in the range of 12–40 kJ/mol suggests that both physical and chemical processes occur during the drying process of tomato peels: a physical process described by the moisture loss of the peels and a chemical process described by the degradation of carotenoids with the temperature and time [8]. The value of E_a_ also offers useful information for drying process optimization, especially for the determination of the required energy for the drying process [30].

### 3.3. Drying Kinetic Parameters and Models Validation

Moisture ratio data obtained from the drying experiments at different temperatures were fitted using ten thin-layer drying mathematical models to determine which model adequately fits the experimental data of tomato peels drying with the HAD method to obtain samples with similar final moisture.

The model parameters and statistical indicators such as R^2^, χ^2^, and RMSE values were calculated to evaluate the accuracy of each mathematical model, as presented in Table 2. The most suitable model was chosen based on the highest R^2^ and lowest χ^2^ and RMSE values. The coefficient of determination R^2^ values show a good prediction for all the models for considered drying temperatures (R^2^ values ranged between 0.9590–0.9999). A slight deviation was observed in the area of higher drying temperatures (75 °C) for Fick’s second law of diffusion model, which considers the diffusion coefficient (R^2^ between 0.9432–0.9999).

To test the model’s goodness fitting, the χ^2^ and RMSE values were calculated using Equations (24) and (25) to predict if the moisture ratios calculated with these models have a higher variance. For Fick’s second law of diffusion model, χ^2^ was less than 0.01 for temperatures below 70 °C, and for semi-theoretical models (STM-N and STM-F), χ^2^ was lower than 0.002 for all the drying temperatures.

Two-term and Modified Henderson and Pabis models present good predictions for moisture removal from tomato peels using HAD and temperatures below 75 °C. Also, it was observed that better results were obtained with models such as *Two-term* and *Midili* with four kinetic parameters and Modified Henderson and Pabis with six parameters. Among all the tested models for tomato peel drying kinetics in the 50–75 °C temperature range, the semi-theoretical model, named the Two-term model, best fits the experimental data, with the highest R^2^ and lowest χ^2^ and RMSE values. The model statistical parameters values are R^2^ between 0.9995–0.9999, χ^2^ between 9.4655 *×* 10^−6^–7.7173 *×* 10^−5^, and RMSE between 0.0013–0.0068. For drying other types of tomato samples (slices, pomace), the Two-term model was also designated as the best in predicting the drying kinetics in different temperature ranges such as 40–70 °C [29] and 38–64 °C [49]. It was also reported that the Midili model fits very well the drying experimental data in the temperature range of 35–45 °C [32] and 30–70 °C [22,31].

**Figure 4:**
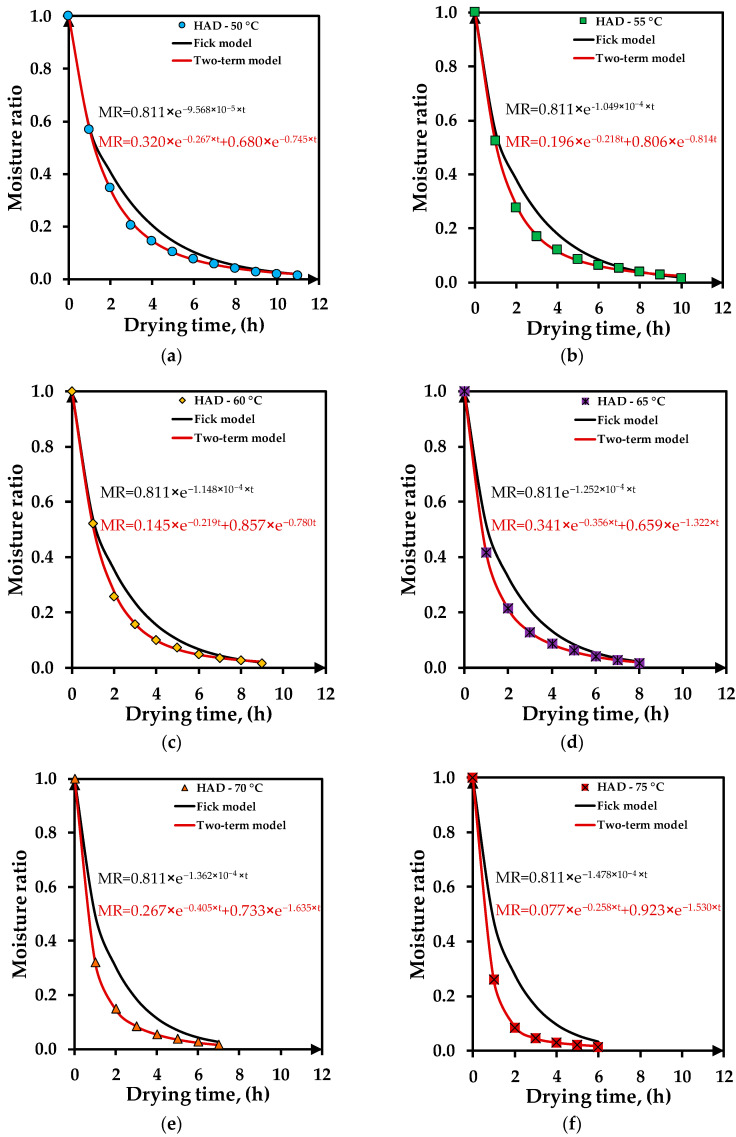
Experimental vs. predicted drying data with Fick’s second law of diffusion and Two-term models at different temperatures: (**a**) 50 °C; (**b**) 55 °C; (**c**) 60 °C; (**d**) 65 °C; (**e**) 70 °C; (**f**) 75 °C.

Figure 4a–f illustrates the variation in drying time of the experimental moisture ratios and predicted values with Fick’s second law of diffusion and Two-term models for temperatures between 50–75 °C. The drying curves have a similar trend to exponential functions decreasing with drying time. At higher temperatures of 70–75 °C, the curves show a faster decrease in moisture ratio. The final moisture ratio was reached at different times according to the drying temperature. At 50 °C, the drying time was the longest, at 11 h, while at 75 °C, the drying time decreased at 6 h. The plots show that the Two-term model best fits the experimental moisture ratios for all drying temperatures. Fick’s second law of diffusion model prediction presents deviation towards the experimental moisture ratios, mostly at higher temperatures.

### 3.4. Carotenoid Degradation

**Figure 5:**
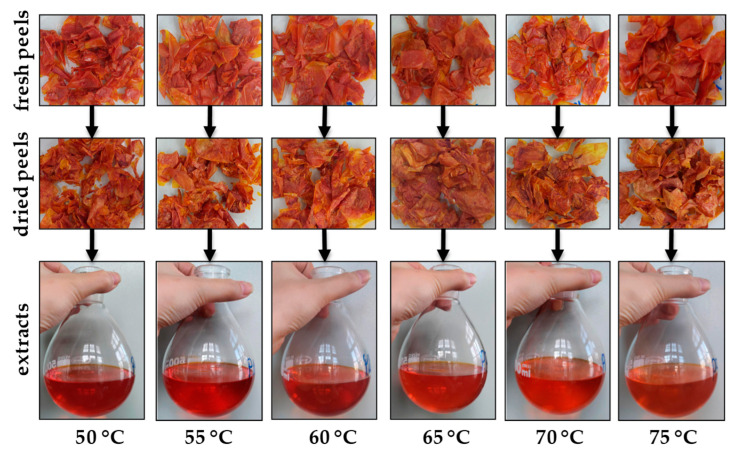
The aspect of the samples in drying and extraction steps.

Figure 5 illustrates the fresh and dried tomato peel samples subjected to HAD drying and the obtained extracts. It can be seen that the peel aspect is similar, with orange–red colors. However, at temperatures higher than 65 °C, the peel color seems to be changed, becoming more orange–brown. This browning reaction is associated with carotenoid degradation during tomato sample drying. This was also observed in other studies at drying temperatures higher than 65–70 °C [23,24]. In what concerns the extracts, their colors are also different according to the drying temperature of the sample. The extracts obtained from dried peels at 50–60 °C have a red color, being more pigmented than the extracts obtained from dried peels at 65–75 °C, with orange colors.

The drying temperature also influences the amount of obtained extracts. Table 3 presents the extraction yields and carotenoid content obtained from dried peels. From dried peels at 50–65 °C, the extraction yield is around 4.58 ± 0.27 g extract/100 g dried peels toward 3.66 ± 0.23 g extract/100 g dried peels at 70–75 °C. The lycopene content of the obtained extracts varies between 31.16 ± 1.11–95.56 ± 3.92 mg/100 g dried peels, while β-carotene levels vary between 47.33 ± 1.54–96.22 ± 1.56 mg/100 g dried peels. These values are in agreement with other studies that reported lycopene concentrations in 100 g of dried tomato peels between 7.14 mg [50], 16 mg [51], 18 mg [48], 23.41 mg [25], 82 mg [52], 110.23 mg [12], 113 mg [53], 119.80 mg [26], 124.45 mg [54], 240 mg [27], 272–288 mg [55], and 50.99–328.88 mg [21]. For β-carotene content in 100 g of dried tomato peels, the concentrations varied between 2.70 mg [12], 2.79 mg [26], 2.89 mg [25], 4.60 mg [53], 13.52 mg [56], and 151 mg [52]. The differences between the carotenoid concentrations in dried tomato peels values may be caused by the tomato variety, the moisture content of the sample, and the drying method and conditions. All of these differences between the samples aspect, the extract colors, the extraction yields, and carotenoid contents indicate the degradation of the peel quality with the temperature, mainly of lycopene, which is responsible for the red color of tomatoes, while β-carotene is associated with the orange color [57].

**Figure 6:**
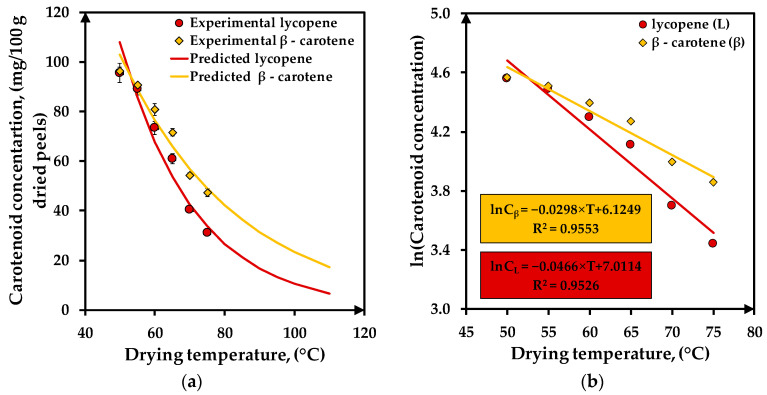
Carotenoid concentrations vs. drying temperature: (**a**) carotenoid concentration experimental/predicted; (**b**) carotenoid degradation model.

Figure 6a presents the variation of lycopene and β-carotene contents from dried tomato peels with the drying temperature. The lycopene and β-carotene degradation degrees from dried tomato peels increase with the drying temperature. Both compounds present high concentrations of around 96 mg/100 g dried peels at 50 °C and low values of 31 mg lycopene and 47 mg β-carotene/100 g dried peels at 75 °C. A small degradation of 5% takes place up to 55 °C, regardless of the carotenoid compound. Between 60–75 °C, the lycopene and β-carotene degradation increases from 21% to 67% and from 16% to 51%, respectively. A similar tendency of lycopene degradation of more than 55% for drying temperatures higher than 65 °C was reported by other studies for tomato peels or slices [8,58,59].

Based on the experimental data regarding the lycopene and β-carotene contents from tomato peels dried at different temperatures, two degradation models (Figure 6b) were formulated using regression analysis. For these models, good values of statistical parameters as R^2^ higher than 0.95 (0.9526 for lycopene and 0.9553 for β-carotene) and low values of χ^2^ (0.0118 for lycopene and 0.0045 for β-carotene) and RMSE (0.0887 for lycopene and 0.0551 for β-carotene) were obtained.

Using the equations of the models illustrated in Figure 6b, the lycopene and β-carotene amounts in dried peels were predicted for the 50–110 °C temperature range, as is presented in Figure 6a. Following the degradation curves, it seems that at 110 °C, the final lycopene and β-carotene contents were 6.59 mg/100 g dried peels and 17.23 mg/100 g dried peels, respectively. These values correspond to a degradation of 94% for lycopene and 83% for β-carotene. The degradation of β-carotene is about 10% higher than lycopene; this behavior is also observed in other studies [57,60].

## 4. Conclusions

The drying temperature significantly influences the moisture removal from tomato peels, the quality of the final dried product, and the energy consumption. For moisture removal, high temperatures lead to quick drying of tomato peels, from 11 h at 50 °C to 6 h at 75 °C, but the quality of the extracts obtained from dried peels is influenced by carotenoid degradation. Significant changes in the lycopene and β-carotene contents were found for drying temperatures between 50 °C and 75 °C: for lycopene from 31.16 ± 1.11 to 95.56 ± 3.92 mg/100 g of dried peels (3 times higher at 50 °C) and for β-carotene from 47.33 ± 1.54 to 96.22 ± 1.56 mg/100 g of dried peels (2 times higher 50 °C). Specific energy requirements between 56.60 ± 0.51–63.00 ± 0.67 kWh/kg peels were calculated for drying tomato peels in the 50–75 °C temperature range.

The behavior of moisture in time for tomato peel drying was formulated using a mathematical model based on Fick’s second law of diffusion and nine semi-theoretical models to fit experimental drying data. Kinetic parameters were determined, and the model goodness was evaluated using statistical coefficients. The results showed that the diffusivity coefficients and the activation energy for Fick’s second law of diffusion model are in agreement with literature data, and the Two-term model accurately predicts the tomato peel behavior during the hot-air drying process on a temperature range of 50–75 °C. For carotenoid degradation, two models were formulated based on experimental data and validated by statistical analysis. Increasing the drying temperature from 50 °C to 110 °C, a degradation of 94% for lycopene and 83% for β-carotene were obtained.

Using the hot-air drying method, the recommended drying temperature for Rila tomato peels is 50 °C to avoid carotenoid degradation with a specific energy consumption of 56.60 ± 0.51 kWh/kg tomato peels.

## Figures and Tables

**Table 2:** Thin-layer mathematical models coefficients and statistical parameters for the HAD drying of tomato peels.

Model	Temperature, (°C)	Model Constants	R^2^	χ^2^	RMSE
Fick’s second law of diffusion	50	(8/π^2^) = 0.8106; −(π^2^ *×* D_eff_)/4/(L/2)^2^) = −9.5680 *×* 10^−5^ D_eff_ = 1.0074 *×* 10^−9^	0.9914	0.0017	0.0377
	55	(8/π^2^) = 0.8106; −(π^2^ *×* D_eff_)/4/(L/2)^2^) = −1.0493 *×* 10^−4^ D_eff_ = 1.0226 *×* 10^−9^	0.9841	0.0030	0.0496
	60	(8/π^2^) = 0.8106; −(π^2^ *×* D_eff_)/4/(L/2)^2^) = −1.1475 *×* 10^−4^ D_eff_ = 1.1642 *×* 10^−9^	0.9886	0.0025	0.0444
	65	(8/π^2^) = 0.8106; −(π^2^ *×* D_eff_)/4/(L/2)^2^) = −1.2517 *×* 10^−4^ D_eff_ = 1.2441 *×* 10^−9^	0.9805	0.0048	0.0610
	70	(8/π^2^) = 0.8106; −(π^2^ *×* D_eff_)/4/(L/2)^2^) = −1.3618 *×* 10^−4^ D_eff_ = 1.3712 *×* 10^−9^	0.9590	0.0119	0.0944
	75	(8/π^2^) = 0.8106; −(π *×* D_eff_)/4/(L/2)^2^) = −1.4780 *×* 10^−4^ D_eff_ = 1.5350 *×* 10^−9^	0.9432	0.0207	0.1217
Newton	50	k = 0.5096	0.9974	0.0017	0.0377
	55	k = 0.5989	0.9964	0.0007	0.0251
	60	k = 0.6271	0.9977	0.0004	0.0197
	65	k = 0.7691	0.9948	0.0010	0.0300
	70	k = 1.0250	0.9961	0.0009	0.0278
	75	k = 1.2920	0.9989	0.0004	0.0180
Page	50	k = 0.5854; n = 0.8494	0.9992	0.0001	0.0084
	55	k = 0.6901; n = 0.8143	0.9980	0.0002	0.0133
	60	k = 0.6904; n = 0.8661	0.9983	0.0002	0.0127
	65	k = 0.8978; n = 0.7331	0.9996	0.0001	0.0063
	70	k = 1.1523; n = 0.6865	0.9998	0.0001	0.0047
	75	k = 1.3681; n = 0.7399	0.9994	0.0001	0.0090
Modified Page	50	k = 0.5324; n = 0.8494	0.9992	0.0001	0.0084
	55	k = 0.6341; n = 0.8143	0.9980	0.0002	0.0133
	60	k = 0.6519; n = 0.8661	0.9983	0.0002	0.0127
	65	k = 0.8633; n = 0.7331	0.9996	0.0001	0.0063
	70	k = 1.2294; n = 0.6865	0.9998	0.0000	0.0047
	75	k = 1.5274; n = 0.7399	0.9994	0.0001	0.0090
Henderson and Pabis	50	a = 0.9817; k = 0.5002	0.9978	0.0004	0.0190
	55	a = 0.9846; k = 0.5897	0.9966	0.0007	0.0242
	60	a = 0.9907; k = 0.6215	0.9978	0.0005	0.0192
	65	a = 0.9835; k = 0.7568	0.9951	0.0011	0.0289
	70	a = 0.9912; k = 1.0173	0.9961	0.0010	0.0274
	75	a = 0.9976; k = 1.2897	0.9989	0.0004	0.0179
Modified Henderson and Pabis	50	a = 0.0006; k = 0.0026; b = 0.6443; g = 0.7703; c = 0.3563; h = 0.2814	0.9998	0.0001	0.0044
	55	a = 0.0060; k = 0.0021; b = 0.8061; g = 0.8142; c = 0.1956; h = 0.2181	0.9996	0.0001	0.0080
	60	a = 0.0267; k = 0.0022; b = 0.0238; g = 4.2794; c = 0.9496; h = 0.6675	0.9990	0.0002	0.0093
	65	a = 0.0003; k = 0.0021; b = 0.3407; g = 0.3562; c = 0.6593; h = 1.3215	0.9999	0.0001	0.0023
	70	a = 0.0019; k = 0.0020; b = 0.7209; g = 1.6571; c = 0.2772; h = 0.4242	0.9999	0.0001	0.0013
	75	a = 0.0159; k = 0.0021; b = 0.6377; g = 1.8397; c = 0.3473; h = 0.8840	0.9999	0.0001	0.0036
Midili	50	a = 1.0008; k = 0.5787; n = 0.8807; b = 0.0011	0.9994	0.0001	0.0070
	55	a = 0.9929; k = 0.6378; n = 0.9504; b = 0.0030	0.9982	0.0002	00123
	60	a = 0.9978; k = 0.6635; n = 0.9615; b = 0.0027	0.9988	0.0002	0.0103
	65	a = 1.0004; k = 0.8943; n = 0.7594; b = 0.0011	0.9997	0.0001	0.0056
	70	a = 1.0001; k = 1.1489; n = 0.7230; b = 0.0015	0.9999	0.0001	0.0032
	75	a = 1.0001; k = 1.3649; n = 0.8384; b = 0.0030	0.9998	0.0001	0.0050
Logarithmic	50	a = 0.9652; k = 0.5522; b = 0.0276	0.9987	0.0001	0.0105
	55	a = 0.9621; k = 0.6677; b = 0.0345	0.9985	0.0002	0.0113
	60	a = 0.9713; k = 0.6841; b = 0.0278	0.9990	0.0001	0.0094
	65	a = 0.9552; k = 0.8727; b = 0.0387	0.9973	0.0004	0.0157
	70	a = 0.9626; k = 1.1535; b = 0.0343	0.9980	0.0003	0.0140
	75	a = 0.9757; k = 1.4042; b = 0.0239	0.9997	0.0001	0.0056
Two-term	50	a = 0.3204; k_1_ = 0.2673; b = 0.6800; k_2_ = 0.7452	0.9998	0.0001	0.0043
	55	a = 0.1957; k_1_ = 0.2180; b = 0.8062; k_2_ = 0.8145	0.9996	0.0001	0.0056
	60	a = 0.1455; k_1_ = 0.2194; b = 0.8568; k_2_ = 0.7801	0.9995	0.0001	0.0068
	65	a = 0.3408; k_1_ = 0.3562; b = 0.6593; k_2_ = 1.3215	0.9999	0.0001	0.0023
	70	a = 0.2667; k_1_ = 0.4049; b = 0.7333; k_2_ = 1.6353	0.9999	0.0001	0.0013
	75	a = 0.0769; k_1_ = 0.2581; b = 0.9232; k_2_ = 1.5304	0.9999	0.0001	0.0020
Two-termexponential	50	a = 0.3668; k = 1.0225	0.9994	0.0001	0.0074
	55	a = 0.3621; k = 1.2147	0.9984	0.0002	0.0135
	60	a = 0.4161; k = 1.0962	0.9988	0.0001	0.0109
	65	a = 0.3055; k = 1.8999	0.9985	0.0002	00154
	70	a = 0.3227; k = 2.3944	0.9985	0.0003	0.0166
	75	a = 0.3804; k = 2.5416	0.9993	0.0001	0.0120

k, k_1_, k_2_, g, h—the drying constants (s^−1^); a, b, c, n—the model constants (dimensionless); D_eff_—the effective diffusivity coefficient (m^2^/s); L—the slab thickness (m).

**Table 3:** Extraction yield (mg/100 g extract ± SD) and carotenoids recovery (mg/100 g dried peels ± SD) from dried tomato peels.

Temperature, (°C)	Yield *	Lycopene *	β-Carotene *
50	4.80 ± 0.17 ^a^	95.56 ± 3.92 ^b^	96.22 ± 1.56 ^c^
55	4.84 ± 0.14 ^a^	88.94 ± 2.30 ^b^	90.70 ± 0.86 ^c^
60	4.33 ± 0.05 ^a^	73.39 ± 2.57 ^b^	80.89 ± 2.39 ^c^
65	4.37 ± 0.11 ^a^	60.87 ± 1.99 ^b^	71.59 ± 1.39 ^c^
70	3.48 ± 0.08 ^a^	40.42 ± 0.84 ^b^	54.32 ± 1.01 ^c^
75	3.84 ± 0.17 ^a^	31.16 ± 1.11 ^b^	47.33 ± 1.54 ^c^

* means ± SD followed by a letter (a–c) indicate that there were no statistically significant differences between the yield or carotenoid concentrations for sets variances with the same superscript letter according to Hartley’s Fmax test (*p* < 0.05) at the α = 0.05 level of significance.

## Data Availability

Data is contained within the article.

## References

[1] Al Maiman S.A., Albadr N.A., Almusallam I.A., Al-Saad M.J., Alsuliam S., Osman M.A., Hassan A.B. (2021). The potential of exploiting economical solar dryer in food preservation: Storability, physicochemical properties, and antioxidant capacity of solar-dried tomato (*Solanum lycopersicum*) Fruits. Foods.

[2] Li L., Yang C., Zhang J., Zhang L. (2022). Study on the drying technology of tomato pulp with phytoene, phytofluene and lycopene retention as inspection indexes. Foods.

[3] Aniceto J.P.S., Rodrigues V.H., Portugal I., Silva C.M. (2022). Valorization of tomato residues by supercritical fluid extraction. Processes.

[4] Gorecka D., Wawrzyniak A., Jedrusek-Golinska A., Dziedzic K., Hamulka J., Kowalczewski P.L., Walkowiak J. (2020). Lycopene in tomatoes and tomato products. Open Chem..

[5] Azeez L., Adebisi S.A., Oyedeji A.O., Adetoro R.O., Tijani K.O. (2019). Bioactive compounds’ contents, drying kinetics and mathematical modelling of tomato slices influenced by drying temperatures and time. J. Saudi Soc. Agric. Sci..

[6] Bhatkar N.S., Shirkole S.S., Mujumdar A.S., Thorat B.N. (2021). Drying of tomatoes and tomato processing waste: A critical review of the quality aspects. Dry. Technol..

[7] Darwish S.M.I., Abd El-Hakim H.I., Abd EL-Rahman M.A.M., Megali H.K.H. (2019). Extraction and utilization of tomato peels lycopene as antioxidant and natural colorants in beef burger. J. Food Dairy Sci..

[8] Toledo Y.S., Macias R.C., Cardenas B.Z., Viera L.C. (2022). Tomato skin drying: Mathematical modeling and effect on lycopene content extracted with *Moringa oleifera* oil. AFINAE.

[9] Madia V.N., De Vita D., Ialongo D., Tudino V., De Leo A., Scipione L., Di Santo R., Costi R., Messore A. (2021). Recent advances in recovery of lycopene from tomato waste: A potent antioxidant with endless benefits. Molecules.

[10] Lazzarini C., Casadei E., Valli E., Tura M., Ragni L., Bendini A., Toschi T.G. (2022). Sustainable drying and green deep eutectic extraction of carotenoids from tomato pomace. Foods.

[11] Umbreen H., Javid M., Riaz M., Nisa M., Zia-Ul-Haq M., Dewanjee S., Riaz M. (2021). Chapter 11—Metabolism of carotenoids. Carotenoids: Structure and Function in the Human Body.

[12] Rizk E.M., El-Kady A.T., El-Bialy A.R. (2014). Characterization of carotenoids (lyco-red) extracted from tomato peels and its uses as natural colorants and antioxidants of ice cream. Ann. Agric. Sci..

[13] Escoto D.F., Ramborger B.P., Gaer M.C., Rodriguez D.T., Denardin E.L.G., Roehrs R., Roehrs M., Bailey J.R. (2015). Chapter 3—Lycopene extraction and analysis. Lycopene food Sources, Potential Role in Human Health and Antioxidant Effects.

[14] Mamouda M.N.A., Saidou M., Makinta B. (2019). Drying kinetics of tomato, okra, potato and mango in a forced-convective solar tunnel dryer. Int. J. Sustain. Green Energy.

[15] Younis M., Abdelkarim D., El-Abdein A.Z. (2018). Kinetics and mathematical modeling of infrared thin-layer drying of garlic slices. Saudi J. Biol. Sci..

[16] Perazzini H., Freire F.B., Freire F.B., Freire J.T. (2016). Thermal treatment of solid wastes using drying technologies: A review. Dry. Technol..

[17] Farooq S., Rather S.A., Gull A., Ganai A.A., Masoodi F.A., Wani S.M., Ganaie T.A. (2020). Physicochemical and nutraceutical properties of tomato powder as affected by pretreatments, drying methods, and storage period. Int. J. Food Prop..

[18] Tahmasebi M., Emam-Djomeh Z. (2021). Lycopene degradation and color characteristics of fresh and processed tomatoes under the different drying methods: A comparative study. Chem. Pap..

[19] Hussein J.B., Oke M.O., Ajetunmobi R.I., Agboola F.F. (2022). Modelling the drying properties of tomato in a hot-air dryer using hybrid ANN-GA technique. J. App. Sci..

[20] Farcas A.C., Socaci S.A., Michiu D., Biris S., Tofana M. (2019). Tomato waste as a source of biologically active compounds. Bull. Univ. Agric. Sci. Vet. Med. Cluj-Napoca. Food Sci. Technol..

[21] Popescu M., Iancu P., Plesu V., Todasca M.C., Bildea C.S. (2019). Effect of different drying processes on lycopene recovery from tomato peels of Crystal variety. U.P.B. Sci. Bull. Ser. B..

[22] Khama R., Aissani-Benissad F., Alkama R., Fraikin L., Leonard A. (2022). Modeling of drying thin layer of tomato slices using solar and convective driers. Agric. Eng. Int. CIGR J..

[23] Mokhtarian M., Majd M.H., Garmakhany A.D., Zaerzadeh E. (2021). Predicting the moisture ratio of dried tomato slices using artificial neural network and genetic algorithm modeling. Res. Innovation Food Sci. Technol..

[24] Moreno D.C., Diaz-Moreno A.C. (2017). Effect of air drying process on the physicochemical, antioxidant, and microstructural characteristics of tomato cv. Chonto. Agron. Colomb..

[25] Albanese D., Adiletta G., D’Acunto M., Cinquanta L., Di Matteo M. (2014). Tomato peel drying and carotenoids stability of the extracts. Int. J. Food Sci.Technol..

[26] Kehili M., Kammlott M., Choura S., Zammel A., Zetzl C., Smirnova I., Allouche N., Sayadi S. (2017). Supercritical CO_2_ extraction and antioxidant activity of lycopene and β-carotene-enriched oleoresin from tomato (*Lycopersicum esculentum L.*) peels by-product of a tunisian industry. Food Bioprod. Process..

[27] Hatami T., Meireles M.A.A., Ciftici O.N. (2018). Supercritical carbon dioxide extraction of lycopene from tomato processing by-products: Mathematical modelling and optimization. J. Food Eng..

[28] Onwude D.I., Hashim N., Janius R.B., Nawi N.M., Abdan K. (2016). Modeling the thin-layer drying of fruits and vegetables: A review. Compr. Rev. Food Sci. Food Saf..

[29] Famurewa J.A.V., Begbaaji O.J. (2018). Mathematical modelling of drying kinetics of tomatoes using cabinet dryer. Ife J. Sci. Technol..

[30] Guine R.P.F., Lima M.J. (2020). Study of the drying kinetics and calculation of mass transfer properties in hot air drying of *Cynara cardunculus*. Open Agric..

[31] Badaoui O., Hanini S., Djebli A., Haddad B., Benhamou A. (2019). Experimental and modelling study of tomato pomace waste drying in a new solar greenhouse: Evaluation of new drying models. Renew. Energy.

[32] Coskun S., Doymaz I., Tunckal C., Erdogan S. (2017). Investigation of drying kinetics of tomato slices dried by using a closed loop heat pump dryer. Heat Mass Transf..

[33] Nwabuka N.R., Chukwuezie O.C., Asonye G.U., Asoegwu S.N. (2020). Influence of process parameters on the energy requirements and dried sliced tomato quality. Eng. Rep..

[34] Crank J. (1975). The Mathematics of Diffusion.

[35] Bruce D.M. (1985). Exposed-layer barley drying, three models fitted to new data up to 150 °C. J. Agric. Eng. Res..

[36] Page G.E. (1949). Factors Influencing the Maximum Rates of Air Drying Shelled Corn in thin Layers. Master’s Thesis.

[37] White G.M., Ross I.J., Ponelert R. (1981). Fully exposed drying of popcorn. Trans. ASAE..

[38] Henderson S.M., Pabis S. (1961). Grain drying theory. II. Temperature effects on drying coefficients. J. Agric. Eng. Res..

[39] Karathanos V.T. (1999). Determination of water content of dried fruits by drying kinetics. J. Food Eng..

[40] Henderson S.M. (1974). Progress in developing the thin layer drying equation. Trans. ASAE..

[41] Sharaf-Eldeen Y.I., Blaisdell J.L., Hamdy M.Y. (1980). A model for ear corn drying. Trans. ASAE.

[42] Midilli A., Kucuk H., Yapar Z. (2002). A new model for single layer drying. Dry. Technol..

[43] Togrul I.T., Pehlivan D. (2002). Mathematical modelling of solar drying of apricots in thin layers. J. Food Eng..

[44] Popescu M., Iancu P., Plesu V., Bildea C.S., Todasca M.C. (2022). Different spectrophotometric methods for simultaneous quantification of lycopene and β-carotene from a binary mixture. LWT-Food Sci. Technol..

[45] Konieczka P., Namiesnik J. (2009). Quality Assurance and Quality Control in the Analytical Chemical Laboratory: A Practical Approach.

[46] Shi J., Jun Xue S., Jiang Y., Ye X. (2010). Supercritical-fluid extraction of lycopene from tomatoes. Separation, Extraction and Concentration Processes in the Food, Beverage and Nutraceutical Industries.

[47] Nobre B.P., Palavra A.F., Pessoa F.L.P., Mendes R.L. (2009). Supercritical CO_2_ extraction of trans-lycopene from portuguese tomato industrial waste. Food Chem..

[48] Lavecchia R., Zuorro A. (2008). Improved lycopene extraction from tomato peels using cell-wall degrading enzymes. Eur. Food. Res. Technol..

[49] Mariem S.B., Mabrouk S.B. (2014). Drying characteristics of tomato slices and mathematical modeling. Int. J. Energy Eng..

[50] Meegahawaththa W.K., Singhalage I.D., Mudannayake D.C. (2020). Tomato (*Lycopersicon esculentum L*.) peel powder as a source of natural antioxidant and a colorant in stirred yoghurt. Food Life.

[51] Vallecilla-Yepez L., Ciftici O.N. (2018). Increasing cis-lycopene content of the oleoresin from tomato processing byproducts using supercritical carbon dioxide. LWT-Food Sci. Technol..

[52] Machmudah S., Zakaria, Winardi S., Sasaki M., Goto M., Kusumoto N., Hayakawa K. (2012). Lycopene extraction from tomato peel by-product containing tomato seed using supercritical carbon dioxide. J. Food Eng..

[53] Topal U., Sasaki M., Goto M., Hayakawa K. (2006). Extraction of lycopene from tomato skin with supercritical carbon dioxide: Effect of operating conditions and solubility analysis. J. Agric. Food Chem..

[54] Kehili M., Sayadi S., Frikha F., Zammel A., Allouche N. (2019). Optimization of lycopene extraction from tomato peels industrial by-product using maceration in refined olive oil. Food Bioprod. Process..

[55] Zuorro A. (2020). Enhanced lycopene extraction from tomato peels by optimized mixed-polarity solvent mixtures. Molecules.

[56] Calvo M.M., Dado D., Santa-Maria G. (2007). Influence of extraction with ethanol or ethyl acetate on the yield of lycopene, β-carotene, phytoene and phytofluene from tomato peel powder. Eur. Food Res. Technol..

[57] Bac H.S., Yemis O., Ozkan M. (2023). Thermal stabilities of lycopene and β-carotene in tomato pulp and pink grapefruit juice. J. Food Eng..

[58] Sahin H., Aktas F.T., Orak H., Ulger P. (2011). Influence of pretreatments and different drying methods on color parameters and lycopene content of dried tomato. Bulg. J. Agric. Sci..

[59] Abdulahi N., Kabiruyunusa A., Aliyu A. (2020). Kinetics of the thermal degradation of lycopene in tomatoes. Croat. J. Food Sci. Technol..

[60] Demiray E., Tulek Y., Yilmaz Y. (2013). Degradation kinetics of lycopene, b-carotene and ascorbic acid in tomatoes during hot air drying. LWT-Food Sci. Technol..

